# A color discriminating broad range cell staining technology for early detection of cell transformation

**DOI:** 10.4103/1477-3163.58372

**Published:** 2009-12-16

**Authors:** Idit Sagiv, Pavel Idelevich, Ilia Rivkin, Rimona Margalit, Adi Elkeles, Alexander Levitzki

**Affiliations:** Unit of Cellular Signaling, Department of Biological Chemistry, The Hebrew University of Jerusalem, Jerusalem 91904, Israel; 1 Zetiq Technologies LTD, Paz Tower 1, 7^th^ floor, 5-7 Shoham St., Ramat Gan 52521, Israel; 2 Department of Biochemistry, George S. Wise Faculty of Life Sciences, Tel-Aviv University, 69978 Ramat-Aviv, Israel

**Keywords:** Cancer, cell transformation, early detection, HPV, tinctorial difference

## Abstract

**Background:**

Advanced diagnostic tools stand today at the heart of successful cancer treatment. CellDetect^®^ is a new histochemical staining technology that enables color discrimination between normal cells and a wide variety of neoplastic tissues. Using this technology, normal cells are colored blue/green, while neoplastic cells color red. This tinctorial difference coincides with clear morphological visualization properties, mainly in tissue samples. Here we show that the CellDetect^®^ technology can be deployed to distinguish normal cells from transformed cells and most significantly detect cells in their early pre-cancerous transformed state.

**Materials and Methods:**

In tissue culture, we studied the ability of the CellDetect^®^ technology to color discriminate foci in a number of two stage transformation systems as well as in a well defined cellular model for cervical cancer development, using HPV16 transformed keratinocytes.

**Results:**

In all these cellular systems, the CellDetect^®^ technology was able to sensitively show that all transformed cells, including pre-cancerous HPV 16 transformed cells, are colored red, whereas normal cells are colored blue/green. The staining technology was able to pick up: (i) early transformation events in the form of small type 1 foci (non-invasive, not piled up small, with parallel alignment of cells), and (ii) early HPV16 transformed cells, even prior to their ability to form colonies in soft agar. The study shows the utility of the CellDetect^®^ technology in early detection of transformation events.

## BACKGROUND

Cancer development is an evolving, complex multi-step process. Numerous studies have laid the foundation for the understanding of cancer development from the onset of early transformation to the advanced metastatic cancer cells.[1–3] It has also been realized that it is important to identify the pre-cancerous stage and develop technologies for early cancer diagnosis since it is a key to successful clinical management.[4–7] Studying transformation events *in vitro* is an important means for deciphering disease pathways and identification of compounds that induce cell transformation.[8,9] Many tools available today for studying transformation are either disease specific or general. In the first category the tools are in the form of specific biomarkers, immuno-histochemical stains. In the second category are tools that measure a phenotypic trait of transformed cells, such as the ability to form colonies in soft agar, or the ability to form foci in transformation assays.[10–12] We feel that developing a general analytical tool that can discriminate visually between normal and transformed cells, and even detect cells in their early stages of transformation, is an important addition to such methods. In this report we describe a novel technology - the CellDetect^®^ technology, which enables simple color discrimination between normal and a wide variety of transformed cells as well as detecting cells in their early stages of transformation, actually in their pre-cancerous stage. The CellDetect^®^ technology employs natural dyes that are suggested to interact differently with transformed cells whose intracellular pH is more alkaline, than with their normal counterpart.[13–17]

The color discriminating properties of this histochemical stain is mostly reflected in the cells cytoplasm of neoplastic cells that stain red/purple in contrast to non-neoplastic cells, which stain blue/green.

To evaluate the potency of the CellDetect^®^ technology, we studied two different *in vitro* transformation models. One was the comparison between transformed cells and their untransformed parental cells and the other was the examination of a cell system that evolves continuously from the normal stage to the fully transformed stage. The ability to discriminate normal from transformed cells was studied in a well-defined two-stage transformation assay.[8,12] The two-stage transformation system is widely used for studying transformation and screening the transformation potential of various compounds. A good correlation exists between *in vitro* cell transformation and carcinogenesis.[18–20] Foci are scored positive when the foci shows deep basophilic, dense multi layering of cells, random cell orientation at any part of the focus edge, invasion into the surrounding contact-inhibited monolayer, and domination of spindle-shaped cells.[9] The cell system we utilized to study the representation of cancer evolution was based on a cellular model for the progression of cervical cancer. Cervical cancer is initiated by infection of keratinocytes by Human Papilloma Virus (HPV), which leads to cell immortalization, where full transformation and tumor formation occur only 10-20 years later in 5-10% of infected women. During this long duration, cells accumulate mutations, which eventually lead to full transformation. Progression of the infected cells to the tumor stage is associated with risk factors such as smoking[21] and sexual encounters with many partners.[22] The model cellular system that we developed[23–25] consists of cultures of primary human keratinocytes (K) and of HPV16-transformed keratinocytes (HF1 cells) from early (E ∼60 doublings) and late passage (L ∼1000 doublings).[25,26] L cells were treated with benzo-[a]pyrene (BP), a carcinogen present in cigarette smoke. L and BP cells display enhanced growth rate,[24,25] increased ploidy, and chromosomal translocations, whereas BP cells also generate colonies in soft agar. Furthermore, L and BP cells show a striking convergence of gene expression with published data from cervical carcinomas,[27] attesting the biological relevance of this cellular model system to the development of human cervical cancer. SiHa cervical cancer cells represented the fully transformed state.

## METHODS

### Reagents

Chemicals were obtained from the following sources: N-methyl-N'-nitro-N-nitrosoguanidine (MNNG) from Chemos GmbH, Germany, 3-Methylcholantrene (MC) and 12-O-tetradecanoylphorbol-13-acetate (TPA) from Sigma-Aldrich^®^.

Cell culture reagents were purchased as follows: DMEM, HAM/F12, RPMI and antibiotics from Biological Industries Israel Beit Ha'emek Ltd.; FBS from Gibco BRL Life Technologies, Paisley, United Kingdom.; EGF from Peprotech Inc.; insulin; T3; transferrin; cholera toxin and hydrocortisone from Sigma-Aldrich^®^.

### Cells and cell culture

BALB/3T3 A31-1-1 cells were purchased from the Japanese Cancer Research Recources Bank. Cells were grown in Eagle's MEM medium (Biological Industries Ltd., Israel) supplemented with 10% fetal bovine serum (FBS), 2mM L-glutamine, 100 units/ml penicillin and 0.1 mg/ml streptomycin. Frozen stock ampoules of the cells were prepared at passage eight. One ampoule was thawed and used for each experiment. Other media used in transformation experiments were DME/F-12 modified medium (Gibco BRL), ITES – a mixture of 200 *μ*g/ml bovine pancreas insulin, 200 *μ*g/ml human transferring, 12.2 *μ*g/ml ethanolamine and 0.034 *μ*g/ml sodium selenite (all from Sigma-Aldrich^®^). CH3/10T1/2, clone 8 cells (ATCC number CCL-226) were grown in DMEM medium with L-Glutamine (Gibco BRL) supplemented with 10% FBS, 100 units/ml penicillin and 0.1 mg/ml streptomycin. Frozen stock ampoules of the cells were prepared at passage two. One ampoule was thawed and used for each experiment.

Cells culture flasks were purchased from Nunc and 60-mm dishes from Corning.

Primary keratinocytes and HPV16-immortalized cells were maintained in Keratinocyte Growth Medium (KGM): DMEM, 25% Nutrient mixture F-12 (HAM), 10% fetal bovine serum, 5 *μ*g/ml insulin, 0.4 *μ*g/ml hydrocortisone, 0.1 nM cholera toxin, 10 ng/ml epidermal growth factor, 1.8×10^−4^ M adenine, 5 *μ*g/ml transferrin, 2×10^−9^ M T3, 100,000 U/L penicillin, 100 *μ*g/L streptomycin, 0.1 mg/mL amphotericin. Primary keratinocytes were cultured from small biopsy specimens. The HPV16-immortalized keratinocytes had been transfected with the genome of the human papillomavirus, HPV16.[26] Early passage (passage 20, E) represents cells that underwent about 50 doublings after transfection. Late passage (passage 270, L) represents cells that underwent about 1000 doublings. BP cells were derived from L, by benzo[a]pyrene treatment. L cells were treated with benzo[a]pyrene (5 *μ*M) for 30 days to establish a clone able to form colonies in soft agar. SiHa cells were kindly provided to us by Professor S. Rosenbaum-Mitrani (Hadassah Hospital, Jerusalem, Israel). SiHa cells were grown in RPMI containing 10% FBS and 100,000 U/L penicillin 100 *μ*g/L streptomycin.

The cell lines were cultured at 37°C in a humidified incubator supplied with a constant flow of 5% CO_2_.

### Soft agar assay

Cells were plated in agar at a density of 5000 cells per well in a 96-well plate in growth medium containing 0.3% agar (50 *μ*l per well), on top of a layer of growth medium containing 1% agar (100 *μ*l per well). 50 *μ*l of growth medium were added on top of the agar. Two weeks after plating, the cells were stained with 3-(4, 5-dimethylthiazol-2-yl)-2,5-diphenyl-tetrazolium bromide (MTT; Sigma) 5 mg/ml diluted 1:5 in the agar for two hours and photographed.

### Cell transformation assays

In the two-stage transformation assay, based mainly on protocol of IARC/NCI/EPA Working Group,[9] the cells were treated with two different carcinogenesis initiators, MNNG for BALB/3T3 A31-1-1 cells[28] and MC for CH3/10T1/2 cells.[3] Both systems use TPA (12-O-tetradecanoylphorbol-13-acetate) as a tumor promoter.

#### BALB/3T3 A31-1-1 cells transformation assay

On day 0, the cells were seeded at 1.5×10^5^ cells/60mm dish in 5 ml of growing Eagle's MEM and incubated for 3 days, during which the cells reached plate sub-confluence. The cells were treated with 0.5 *μ*g/ml MNNG for five hours and seeded into culture dishes to examine viability and malignant transformation. Cytotoxicity test was performed as previously described,[8,9] and calculated as the percentage of cells in the treated group compared to controls (data not shown). MNNG-treated cells were re-plated into 60 mm plastic dishes at a cell density of 5×10^3^ cells/60 mm dish. The dishes were incubated for about three weeks with two medium changes per week. TPA was added at100 ng/ml for one week, after re-plating and the cells were kept in the presence of the promoter for 12 days, thereafter the cells were grown in chemicals free medium for three days. At the end of the experiment, 22 days after re-plating, one series of dishes with cells were fixed with methanol and stained with 2% Giemsa solution, while a second series were air dried for 24 hours then stained then with the CellDetect^®^ kit. Transformation foci of types 2 and 3 were scored according to the criteria described elsewhere.[9]

#### C3H/10T1/2 cells transformation assay

The cells harvested from logarithmically growing stock cultures were plated on day 0 onto 60-mm dishes in 5 ml of DMEM growing medium: 200 cells/dish for cytotoxicity determination and 10^3^ cells/dish for the transformation foci count. After 24 hours, the cultures were treated with 5 *μ*g/ml MC and incubated for 72 hours. Thereafter, the medium was renewed and cells were allowed to grow in fresh DMEM medium. The cytotoxicity test was performed as previously described,[9] and calculated as the percentage of cells in the treated group compared to control treated and untreated cells (data not shown). TPA was added to the MC treated cells on day 11, and plates were incubated for 14 days, with two medium changes per week. From day 26 plates were incubated in TPA- medium for additional 17 days. At the end of the experiment, plates were air-dried for 24 hours and stained with CellDetect^®^ kit. The entire experiment lasted six weeks (total culture time). Transformed foci of types 1, 2 and 3 as defined by Reznikoff *et al*,[8] were counted using an inverted microscope.

### Giemsa Staining

Dishes were fixed by cold Methanol (100%) for 10 minutes, thereafter stained by 2% Giemsa solution in distillated water for 33 min. The plates were then washed in distillated water and air-dried for 30 minutes.

### CellDetect^®^ kit

Staining with the CellDetect^®^ kit (Zetiq Technologies Ltd., Ramat Gan Israel) was performed according to manufacture guidelines. The details of the protocol and the formulation of the stains are currently proprietary information.

## RESULTS

### Two-stage transformation

The two-stage transformation assay is a well recognized system to measure transformation induced by chemical mutagens,[8,9,28–30] We wished to explore the potential utility of using the CellDetect^®^ kit to stain cells treated by mutagens, and measure whether the kit can color discriminately the foci generated by transformation events. We used two independent cell lines well characterized for such assays,[8,9] utilizing two independent mutagens, probing whether the color discrimination is a general phenomenon.

#### Experiments were performed as detailed, and foci scoring were performed according to established criteria[8,9] briefly:

For the cell line BALB/3T3 A31-1-1, type 3 foci hold the following properties: dense, multilayered, basophilic, random orientation of focus edge; invasion into the monolayer, transformed cells are predominantly spindle-shaped. Type 2 foci are distinguishable from type 3 foci primarily by their more ordered and defined edge. Type 2 foci also show dense cells features, multilayered but less basophilic than type 3.

C3H/10T1/2 Type 2 foci are characterized by massive piling up into virtually opaque multilayers; the cells are only moderately polar. Hence criss-crossing is not pronounced. Type 3 is composed of highly polar, fibroblastic, multilayered criss-crossed arrays of densely stained cells.

Each of the experiments had proper controls to illustrate the additive effect of the two-stage transformation. The controls were composed of cells with no treatment (negative control group), initiator only (mutagen), TPA only, and initiator + TPA (treatment group). Within each group, four independent plates were prepared.

In the first experiment, two complete sets were prepared. One set was stained with the Giemsa stain and the other set with the CellDetect^®^ kit. In both staining sets, only type 3 foci were scored, as recommended.[8] The results of foci formation for the CellDetect^®^ set are summarized in Table 1.

**Table 1:** Foci formation as observed in CellDetect^®^ staining of BALB/3T3 cells

Initiating treatment	Promoting treatment	Total No. of foci	No. of dishes with foci/No. of dishes examined	Foci/dish
None	None	1	1/4	0.25
None	TPA	3	3/4	0.75
MNNG	None	2	2/4	0.5
MNNG	TPA	18	4/4	4.5

Treatment with both mutagen and TPA largely enhances type 3 foci formation

**Figure 1:**
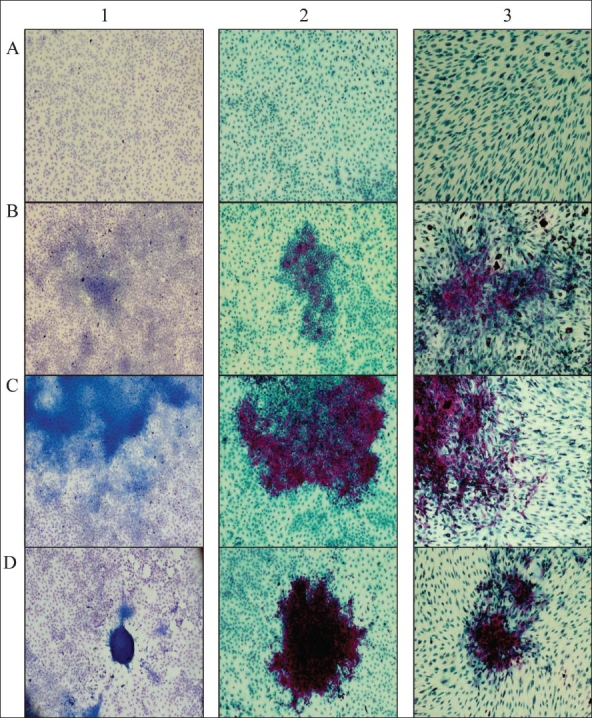
CellDetect^®^ staining of two-stage transformation assay. Giemsa (column 1) and CellDetect^®^ (columns 2 and 3) staining of BALB/3T3 A31-1-1 cells (columns 1 and 2), CH3/10T1/2 cells in column 3, A-normal; B-foci type 1; C-foci type 2; D-foci type 3.

The CellDetect^®^ scoring was similar to the ones obtained after counting type 3 foci in the Giemsa stained set [Figure 1, column 1]. As seen in Table 1, treatment with both mutagen and TPA largely enhances foci formation. These results are as expected, and confirm that this is a well established cell line model. To further validate these results, we repeated the experiment under identical conditions - staining only with the CellDetect^®^ kit to obtain similar results with as many as 54 type 3 foci counted in all the dishes. All the foci were stained red, while normal untransformed monolayer cells were stained green. The striking color discrimination between normal monolayer cells and transformed foci is shown in Figure 1.

To extend and generalize the color discrimination phenomena as seen when staining with the CellDetect^®^, we performed another cell transformation assay, using a different cell line and different initiator mutagen. When using this cell line for transformation assay, we counted type 2 and type 3 foci.[29] The results of foci formation in the experiment with C3H/10T1/2 cells are summarized in Table 2.

**Table 2:** Foci formation as observed in CellDetect^®^ staining of C3H/10T1 cells

Promoting treatment	Initiating treatment	Total No. of foci	No. of dishes with foci/No. of dishes examined	Type 3 foci/dish	Type 2 foci/dish
None	None	0	0/4	0	0
TPA	None	3	2/4	0	0.75
None	MC	7	4/4	0	1.75
TPA	MC	12	4/4	1.75	1.25

Treatment with both mutagen and TPA largely enhances foci formation. The treatment also increases ratio of type 3 foci

Table 2 shows that treatment with both mutagen and TPA largely enhances foci formation. The treatment also increases ratio of type 3 foci. Representative pictures from this experiment are shown in Figure 1.

### Cellular model for cervical cancer evolution

**Figure 2:**
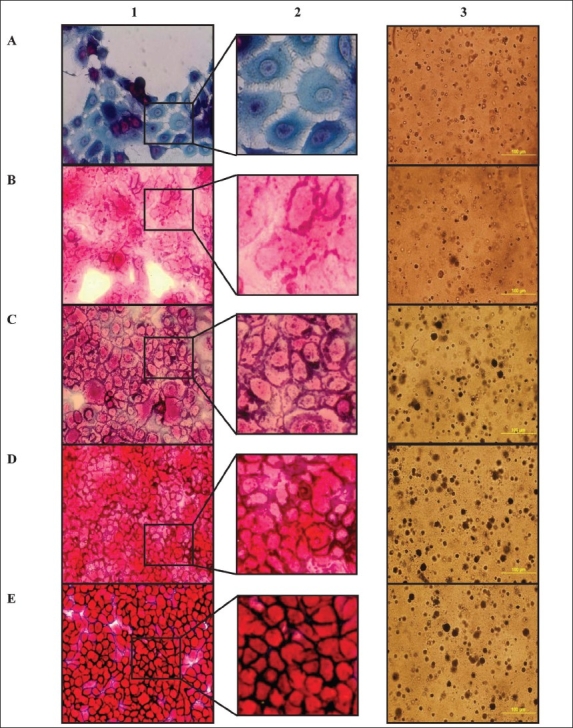
Progression towards a cancerous phenotype. Keratinocytes (A), Early HF1 (B), Late HF1 (C), BP (D) and SiHa cells (E) were stained with CellDetect^®^ kit (columns 1 and 2), and were subjected to colony formation assay on Soft agar (column 3).

To explore the ability of the CellDetect^®^ technology to color-discriminate early transformation events, we utilized a well-defined cellular transformation model.[23,24,31] Normal Keratinocyte cells gave rise to a blue/green color staining in both cytoplasm and nuclei [Figure 2 A1]. HPV16 transformed cells from the different passages, as well as BP transformed cells and the SiHa cell line, stained-red following staining with the CellDetect^®^ technology [Figure 2 B1, C1, D1, E1].

The CellDetect^®^ technology identified by color discrimination early transformation in Early HF1 cells (E), Late (L) and BP HF1.

Primary keratinocytes, early and late HF1 cells were plated in soft agar and grown for two weeks [Figure 2 A3]. In contrast to the primary keratinocytes, immortalized HF1 cells survived in soft agar. E cells survived, but did not proliferate and did not form colonies in soft agar. In contrast, L cells formed both micro-colonies and large colonies in soft agar [Figure 2 C3]. BP cells as well as SiHa formed more colonies, mainly larger ones [Figure 2 D3, E3].

## DISCUSSION

A total of 85 type 3 foci and 15 type 2 foci were counted in all the experiments, all of the total 100-type 2 and type 3 foci contained red-stained cells. This exemplifies the power of the CellDetect^®^ to correctly and sensitively identify independent transformation events.

The detection of type 1 foci is not simple when using Giemsa stain [Figure 1]. The sizes of these foci are small, and in many cases it is hard to discern them from the surrounding monolayer cells. This is one of the reasons why scoring of type 1 foci is not recommended. When we analyzed the CellDetect^®^ stained plates we could easily observe these type 1 foci through the color discrimination properties they exhibited [Figure 1 B2 and B3]: red-stained packed cells in small foci, surrounded by normal blue/green stained monolayer cells. This phenomenon is depicted in Figure 1, showing the potential to use the CellDetect^®^ kit for scoring and analysis of type 1 foci.

Confirming published data, our results also show that the combination of the two transformation stages (initiation and promotion) results in the appearance of many more colonies than in the control plates.[8,9] These data confirm that the experiments agree with known parameters of the two-stage transformation assay. The CellDetect^®^ staining offers sensitive color discrimination for *all* formed foci in the dishes, in two distinct cell lines [Table 2], under the treatment of two different mutagens. The two-stage transformation assay is an important tool, but its major drawback is the difficulty in the distinction of different foci and their scoring.[9] CellDetect^®^ staining holds advantages over Giemsa staining for such assays. This stain provides an easier and a more accurate detection of foci and micro invasions to the monolayer. It has the potential to include type 1 foci scoring, unavailable in current Giemsa staining.

The cervical cancer cellular model consists of transformed HF1 cells that are considered to be pre-cancerous since they do not produce detectable tumors in nude mice. Yet, they have already acquired a plethora of typical features of malignant cells such as enhanced growth rates (generation time decreases continuously from over 24 hours in the primary keratinocytes to 22 hours in E cells and 16 hours in L cells) and gain the ability to survive and grow in soft agar.[23] The morphological features of HF1 cells show features of epithelial to mesenchimal transition (EMT) as the passage moves from keratinocytes to E to L, namely: reduced desmosomes, keratins, adherens junctions and focal adhesions, with the dramatic up-regulation of the transcription factor Twist.[31]

Noticeably, color-shading differences are observed between the different cells in this model. While early transformed E cells are colored light red, late L transformed cells have a more intense red stain, where BP and SiHa cells have a dark red color [Figure 2 E1]. This may suggest a correlation between the transformation stage and color shading or intensity. More work is needed to confirm this exciting observation also in other systems in which one can monitor the continuous transition from the normal to the transformed phenotype.

## COMPETING INTEREST

The authors have declared that no competing interests exist.

## AUTHOR'S PROFILE

**Mrs. Idit Sagiv,** is a PhD student in the laboratory of Professor Alexander Levitzki at the Department of Biological Chemistry at the Hebrew University, Jerusalem Israel. Her interests include the development of proliferative diseases such as cancer and the induction of specific immunological tolerance for the treatment of immunological diseases and transplant rejection.

**Dr. Pavel Idelevich,** trained in internal medicine and pathology at Saratov State Medical Institute, and the Soroka Medical Center in Beersheva, where he gained clinical experience training as a medical doctor in GI and urology units. He served as a researcher in the Immunology Department at the Weizmann Institute of Science. An experienced pathologist, Dr. Idelevich has played a central role in the development of Zetiq's staining technology.

**Prof. Alexander Levitzki,** obtained his M.Sc. degree in chemistry in 1963 from the Hebrew University of Jerusalem, and his Ph.D. in biophysics and biochemistry in 1968 from the Weizmann Institute of Science. After postdoctoral studies with Daniel E Koshland Jr, he continued his research on enzyme cooperativity at the Weizmann Institute. He has been at the Hebrew University since 1974. His laboratory performed pioneering studies on G-protein-coupled signalling and developed the first tyrosine-kinase inhibitors. Other research interests include innovative methods of using double-stranded RNA to kill cancer cells and *in vitro* modelling of cancer development. He has authored over 300 publications and dozens of patents. He was awarded the Wolf Prize for 2005 in Medicine, for his role in developing signal-transduction therapy.

**Mr. Ilia Rivkin,** a Ph.D. student at Tel Aviv University, holds a M.Sc. in Biochemistry from Tel Aviv University and B.Sc in Biotechnology from Braude Academic College of Engineering. Mr. Rivkin has research and practical experience in fields of chemistry and biotechnology including cell-based assay development, HTS robotic automatization and establishing of drug-carrier systems for cancer treatment. Before joining Zetiq as performance manager, Mr. Rivkin served as laboratory and research assistant at Haifa Chemicals and Kinneret Limnological Laboratories.

**Prof. Rimona Margalit,** is a faculty member at Tel Aviv University, holds a PhD from Tel Aviv University, completed a three-year post-doctoral fellowship in Membrane Biophysics at UCLA and has been on sabbaticals in Pharmaceutical Sciences Departments at Baxter Healthcare Corporation and at Aradigm Corporation, both in the USA. Prof. Margalit is the incumbent of the Simko Chair in Breast Cancer Research, and her areas of interest and expertise encompass membrane biophysics, drug delivery, nanoparticles, cancer, diabetics and Arthritis. She is an inventor of several patented drug delivery technologies and is a founding parent of the Israeli Chapter of the Controlled Release Society.

**Dr. Adi Elkeles,** holds a PhD from Tel Aviv University, an MBA from the College of Management, and completed a three-year post-doctoral fellowship in Molecular Cell Biology at the Weizmann Institute of Science. Dr. Elkeles has significant research and development experience in various fields of biology including cancer, immunology and infectious diseases. He is an experienced executive in the biotech field, specializing in biopharmaceutical development. Before joining Zetiq, Dr. Elkeles served as Drug Development Manager for BiolineRx and as manager at XTL Biopharmaceuticals.
